# Human microbiomes in cancer development and therapy

**DOI:** 10.1002/mco2.221

**Published:** 2023-02-26

**Authors:** Chenglai Xia, Jiyan Su, Can Liu, Zhikai Mai, Shuanghong Yin, Chuansheng Yang, Liwu Fu

**Affiliations:** ^1^ Affiliated Foshan Maternity and Chlid Healthcare Hospital Southern Medical University, Foshan, China; School of Pharmaceutical Sciences, Southern Medical University Guangzhou China; ^2^ Department of Head‐Neck and Breast Surgery Yuebei People's Hospital of Shantou University Shaoguan China; ^3^ State Key Laboratory of Oncology in South China Collaborative Innovation Center for Cancer Medicine; Guangdong Esophageal Cancer Institute; Sun Yat‐sen University Cancer Center Guangzhou People's Republic of China

**Keywords:** cancer therapy, carcinogenesis, microbiome, microorganism, tumor

## Abstract

Colonies formed by bacteria, archaea, fungi, and viral groups and their genomes, metabolites, and expressed proteins constitute complex human microbiomes. An increasing evidences showed that carcinogenesis and disease progression were link to microbiomes. Different organ sources, their microbial species, and their metabolites are different; the mechanisms of carcinogenic or procancerous are also different. Here, we summarize how microbiomes contribute to carcinogenesis and disease progression in cancers of the skin, mouth, esophagus, lung, gastrointestinal, genital, blood, and lymph malignancy. We also insight into the molecular mechanisms of triggering, promoting, or inhibiting carcinogenesis and disease progress induced by microbiomes or/and their secretions of bioactive metabolites. And then, the strategies of application of microorganisms in cancer treatment were discussed in detail. However, the mechanisms by which human microbiomes function are still poorly understood. The bidirectional interactions between microbiotas and endocrine systems need to be clarified. Probiotics and prebiotics are believed to benefit human health via a variety of mechanisms, in particular, in tumor inhibition. It is largely unknown how microbial agents cause cancer or how cancer progresses. We expect this review may open new perspectives on possible therapeutic approaches of patients with cancer.

## Graphical abstract

Several microbes, such as *Fn*, *Helicobacter pylori*, *Fusobacterium*, *Staphylococcus aureus*, *Peptostreptococcus anaerobius*, and *Porphyromonas gingivalis*, promote cancer progression. Other microbes, such as *Lactobacillus*, *Bifidobacterium*, *Clostridium butyricum*, *Bacillus subtilis*, *Bifidobacteria*, *Lactobacoilli*, *Streptococcus thermophilus*, *Leptotrichia*, *Bacteroides thetaiotaomicron*, and *Faecalibacterium prausnitzii*, can inhibit cancer progression. Possible therapeutic approaches for cancer include fecal microbiome transplantation (FMT), probiotics, synbiotics, antibiotics, oncolytic virus, and nanospheres.

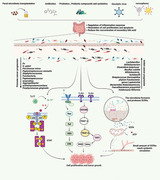

## 1 INTRODUCTION

Approximately 1 × 10^12^ types of microbiomes are known to exist on Earth. Numerous microorganisms live in the gut, the skin, the lungs, and the oral cavity. Additionally, the human microbiome, often called the “hidden organ,” contributes more genetic information than the whole human genome combined.^1^, ^2^, ^3^ Advances in recent years have uncovered a close relationship between the human microbiome and its ability to extract nutrients, process carbohydrates, and create antibodies. There are many possible mechanisms for the influence of the microbiomes on biological processes. Microbiomes play an important role in converting food into energy, nutrition, and bioactive molecules, such as vitamins, lipids, and amino acids. Furthermore, the human microbiomes contribute to mucosal and immune system development, as well as to protection from external pathogens.^4^, ^5^, ^6^ However, the system consists of trillions of microbiomes.

Using bioinformatics and advanced sequencing technologies, scientists are discovering the relationship between the microbiome and disease. Changing environmental conditions can disrupt the balance of microbiomes, resulting in dysregulation of bodily functions and disease.^7^, ^8^, ^9^

There are more cancer deaths worldwide than any other disease.^10^, ^11^, ^12^ The risk factors of cancer are very complex, and environmental factors account for a high proportion of common risk factors. The symbiotic microbiome is a major regulator of carcinogenesis.^10^, ^13^ A comparison of cancer growth in germ‐free mouse models or models where antibiotics are used to deplete the gut microbiome with mouse models maintained on normal feed suggests that there is a strong correlation between cancer growth and the microbiome.^14^, ^15^ Overall, it has been demonstrated that dysbiosis of the microbiome can initiate and progress cancer .

In the 1970s, a pathologist from the Royal Perth Hospital in Australia (J. Robin Warren) found that *Helicobacter pylori* (*H. pylori*) bacteria were frequently observed in the gastric tissue of chronic gastritis patients. Warren and Barry J. Marshall speculated that *H. pylori* may therefore cause gastritis.^16^ To confirm the relationship between this bacteria and chronic gastritis, Marshall underwent an endoscopy to confirm that he was *H. pylori* negative and then infected himself with the bacteria. He then developed gastritis and recorded the development of his disease.^17^ *H. pylori* is considered a common gut microorganism that causes chronic gastritis, gastric ulcers, and gastric atrophy. Additionally, it contributes to gastric cancer development. Approximately half of the population worldwide are infected with *H. pylori*.^18^, ^19^, ^20^

In the early 2000s, microbiomes and cancer have been studied systematically by researchers. A total of seven viruses, two parasites, and one bacteria are human carcinogens, according to the International Agency for Research on Cancer. These include *Epstein–Barr virus* (EBV), *hepatitis B virus*, *hepatitis C virus*, *Kaposi sarcoma herpesvirus*, *human immunodeficiency virus‐1*, *human papillomaviruses* (HPV), *human T‐cell lymphotropic virus Type 1*, *Opisthorchis viverrini* and *Clonorchis sinensis*, *Schistosoma haematobium*, and *H. pylori* ^21^, ^22^ (Table 1). Other fungi also are human carcinogens, such as *Aspergillus flavus*. An increasing number of microbiomes have been identified as promoters but not inducers of cancer. The gut microbiomes is a collection of microorganisms in the human gastrointestinal tract, which makes up 70% of all microbiomes in the human system. Bacteria are also found in the skin, oral and nasal cavity, lungs, esophagus, and vaginal area. However, the number of bacteria in the extraintestinal organs constitutes 3% or less of all bodily microbiomes. Gut microbiomes secretions pass into the local tissues and circulatory system, interacting with cancer cells.^21^, ^23^ Other organs such as the lungs,^24^ breasts,^24^, ^25^ skin,^25^ and urogenital system^26^ are also host to microorganisms, indicating that the organ‐specific microbiome may play a role in carcinogenesis through alteration of the local microenvironment via microbiomes and their metabolites. Importantly, secreted microbial products from microbiomes in the gastrointestinal tract, lungs, breasts, and female reproductive tract can enter the circulation, activate immune signals, and participate in the immune response and immune surveillance.^26^, ^27^, ^28^

**TABLE 1:** Human carcinogenic or cancer‐promoting microbiomes in cancer.

Microbes	Cancer
**Carcinogenic microbes**	
Viruses and parasites	
*Epstein‐Barr virus*	Burkitt lymphoma,^41^ virus‐associated T‐cell and NK‐cell lymphomas,^42^ cancer of the nasopharynx,^43^ lymphoepithelioma‐like carcinomas,^44^ cancer of the stomach^45^
*Hepatitis B virus*	Hepatocellular carcinoma,^46^ cholangiocarcinoma,^47^ non‐Hodgkin lymphoma,^48^, ^49^ cancer of the pancreas,^49^ Hodgkin disease^50^
*Hepatitis C virus*	Hepatocellular carcinoma,^51^ gallbladder cancer,^52^ lymphoid malignancies,^53^ leukemias,^53^ cancer of the thyroid^54^
*Kaposi sarcoma herpesvirus*	Kaposi sarcoma,^55^ primary effusion lymphoma,^56^ multiple myeloma^56^
*Human immunodeficiency virus‐1*	Kaposi sarcoma,^57^ non‐Hodgkin lymphoma,^58^ Hodgkin lymphoma,^58^ cervical and anogenital cancers,^59^ the skin cancer,^60^ the conjunctiva cancer,^61^ the lung cancer,^62^ the liver cancer,^63^ the lip cancer,^64^ the head and neck cancer^65^
*Human papillomaviruses*	The cervix cancer,^66^ the vulva cancer,^67^ the vagina cancer,^67^ the penis cancer,^68^ the anus cancer,^68^ the oral cavity cancer,^69^ the oropharynx and tonsil cancer,^70^ the esophagus cancer,^68^ the larynx cancer,^68^ the skin cancer,^71^ the nose and nasal sinuses cancer,^68^ the lung cancer,^72^ the colon and rectum cancer,^68^ the breast cancer,^73^ the ovary cancer,^74^ the prostate cancer,^75^ the urinary bladder and urethra cancer^76^
*Human T‐cell lymphotropic virus Type 1*	T‐cell malignancies,^77^ cutaneous T‐cell lymphoma,^77^ B‐ and T‐cell lymphomas,^78^ nonlymphomatous tumors^79^
*Opisthorchis viverrini and Clonorchis sinensis*	Cholangiocarcinoma,^80^ hepatocellular carcinoma^80^
*Schistosoma haematobium*	The urinary bladder cancer,^81^, ^82^ the female genital tract cancer,^82^ the prostate cancer^83^
Bacteria	
*Helicobacter pylori*	The stomach cancer,^84^ Gastric mucosa‐associated lymphoid tissue (MALT) lymphoma,^85^ the esophagus cancer,^86^ the liver cancer,^87^ the colorectum cancer,^88^ the pancreas cancer,^89^ the lung cancer,^90^ the head and neck cancer,^91^ Childhood leukemia^92^

Cancer‐promoting microbes	Cancer
Bacteria	
*Enterotoxigenic Bacteroides fragili* *Peptostreptococcus anaerobius* *Fusobacteriumnuleatum*	The colorectum cancer^93^, ^94^
*Hemophilus*, *Porphyromonas gingivalis, Prevotella*	The esophagus cancer^95^
*Micromonospra* *Pporphyromonas gingivalis* *Neisseria, Fusobacteria* *Escherichia Coli, Streptococcus faecalis* *Helicobacter hepaticus*	The liver cancer^96^
*Fermentation bacillus, lactobacillus*	The esophagus cancer^97^
*Streptococcus pneumoniae*	The esophagus cancer^97^
*Staphylococcus aureus*	The skin cancer^98^
*Tuberculous bacillus (TB)*	The lung cancer^99^
*Actinomyces, Falclella, Campylobacter*	The urinary bladder cancer^100^
Fungi	
*Aspergillus*, *Malassezia*, *Malasus*, *Pseudospecies*, *Asppersporus*, *Machyus*	The colorectum cancer^101^
Microbe‐induced metabolite	
Deoxycholic acid	The colorectum cancer^102^

Fungi have been used to treat human diseases since ancient times. Many biologically active compounds extracted from fungi are currently the foci of extensive research. Cohort studies conducted independently in China and Europe found that the amount of malasella increased in colorectal cancer (CRC) patients, while the amount of yeast decreased.^29^ The distribution of *Aspergillus*, *Malassezia*, *Malasus*, *Pseudospecies*, *Asppersporus*, and *Machyus* was altered in CRC patients.

**FIGURE 1:**
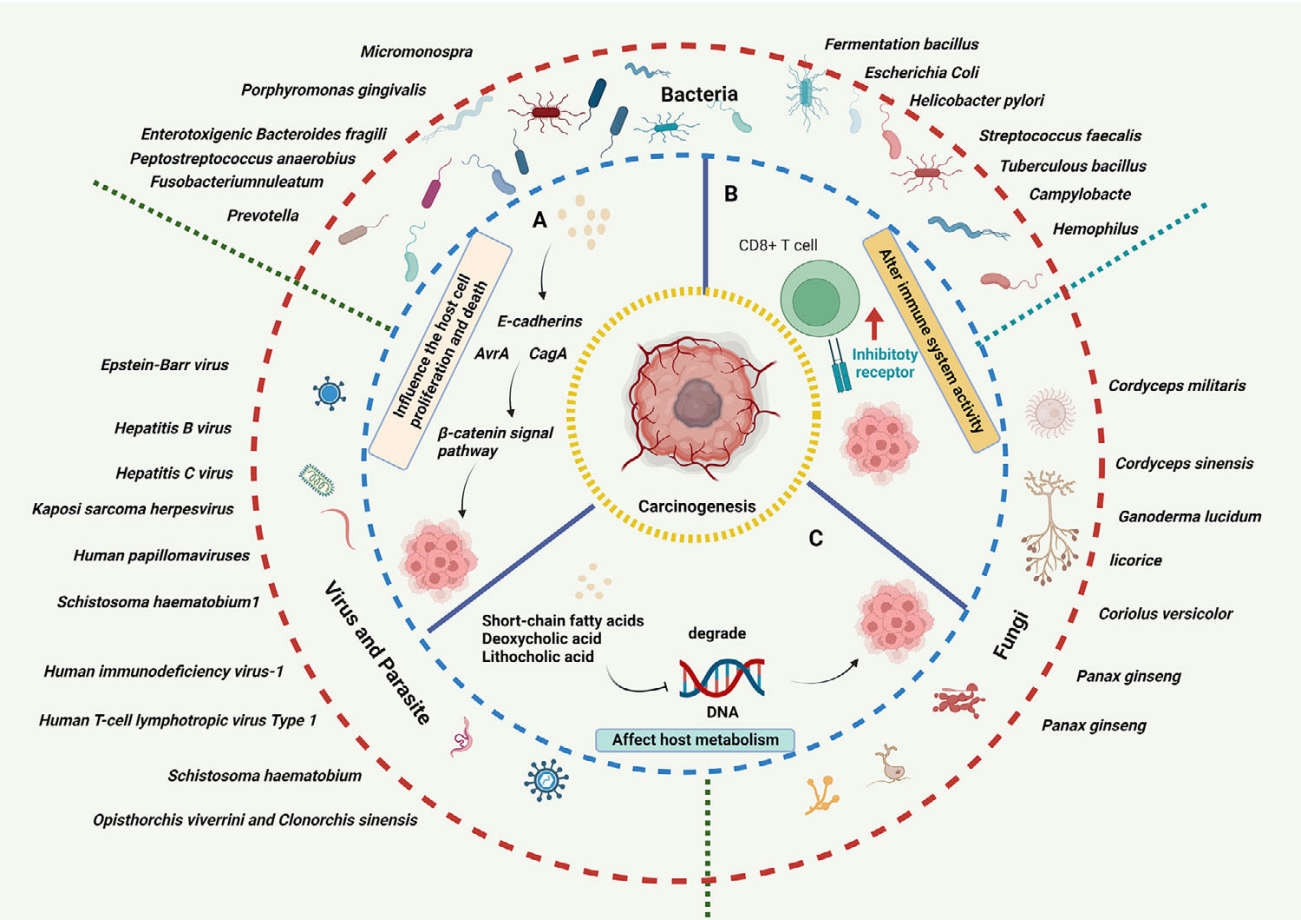
Human carcinogenic microbiomes and cancer. (A) Influence host cell proliferation and death. Microbiomes bind to E‐cadherins, which trigger β‐Catenin activation through polarity changes of barrier disruption or activate the β‐Catenin signaling pathway through CagA or AvrA, leading to dysregulation of cell proliferation. (B) Altering immune system activity. Microbiomes and microbial metabolism bind to inhibitory receptor TIGIT on human immune cells, drive inflammatory responses, and promote cancer immune evasion. (C) Effects on host metabolism. SCFAs, DCA, and LCA, which produced by microbiomes, promote carcinogenesis through host metabolism.

Overall, a number of mechanisms remain to be explored to understand how microbiomes influence the development of cancer, including:

- *Influence on host cell proliferation and death*: Some microbiomes bind to E‐cadherins, which trigger β‐Catenin activation through polarity changes of barrier disruption. Other microbiomes can activate the β‐Catenin signaling pathway through cytotoxin‐associated gene A (CagA) or avirulence gene (AvrA), leading to cell proliferation dysregulation, stem cell‐like growth capability, and loss of polarity.
- *Alteration of immune system activity*: Microbiomes and microbial metabolism can cause cancer by driving inflammatory responses and promoting cancer immune evasion by binding to human natural killers (NKs) and T cells via the inhibitory receptor TIGIT.^30^, ^31^, ^32^ There is evidence that some invasive *Campylobacter* species induce an IL‐18‐driven proinflammatory response that leads to tumor progression.^33^, ^34^
- *Mechanistic effects on host metabolism*: Microbiomes produce short‐chain fatty acids (SCFAs), mainly butyrate, acetate, and propionate, through fiber fermentation.^35^, ^36^, ^37^ This indirectly promotes carcinogenesis through host metabolism and direct destruction of host DNA or the utilization of host superoxide to degrade DNA. Apart from SCFAs, other bacteria metabolites also were involved in carcinogenesis.^38^ A high‐fat diet increases secondary bile acids such as deoxycholic acid (DCA) and lithocholic acid (LCA), which can cause colonic inflammation and cancer^39^, ^40^ (Figure 1).

## 2 HUMAN CARCINOGENIC OR CANCER‐PROMOTING MICROBIOMES

According to the different pathogenic of human microbiomes, we divided the tumor‐causing microorganisms into carcinogenic microorganisms and cancer‐promoting microorganisms. Oncogenic microorganisms can directly induce somatic mutations, promote tumor cell proliferation and tumorigenesis. Cancer‐promoting microorganisms cannot cause tumorigenesis by themselves, but they can secrete cytokines to affect the tumor microenvironment and promote tumorigenesis. In this section, we review the oncogenic microorganisms and cancer‐promoting microorganisms in human physiological systems.

### 2.1 Gut microbiomes and CRC

Increasing evidence has revealed that gastrointestinal microbiome play a critical role in colon cancer carcinogenesis and progression. Kado et al. reported that adenocarcinomas of the ileocecum and cecum were detected in 70% of TCR‐β (−/−)/p53(−/−) mice in the conventionally fed group but not in the germ‐free group, suggesting that intestinal microbiomes play a major role in the development of adenocarcinoma of the colon in this animal model.^103^

Further, an Massachusetts Institute of Technology (MIT) team found that mice infected with *citrobacter murine* were also more likely to develop colon cancer.^104^ In 2003, Susan Erdman's team at MIT showed that *H. pylori* caused colon cancer in immune‐compromised mice.^105^ In 2006, Erdman's team infected a particular mouse model with *H. hepaticus* and continued to observe subsequent changes in the mice. Unexpectedly, many of the mice developed breast cancer, showing that mice infected with *H. hepaticus* are likely to develop breast cancer.^106^ In 2013, Schloss’ team compared tumor‐bearing mice treated with antibiotics prior to the induction of cancer or proinflammatory treatments to tumor‐bearing mice that received only cancer and inflammatory treatments. They found that the size and number of tumors in the antibiotic‐treated mice were significantly smaller than in the control group. In addition, when the researchers transferred microbes from the healthy mice to the antibiotic‐treated or germ‐free mice, the latter's exposure to carcinogens led to more malignant tumor tissue size.^107^ In 2014, Patrick Schloss’ team from the University of Michigan sequenced the 16S ribosomal RNA(16S rRNA) gene from 90 samples of human feces. The samples were provided by patients with colon cancer, patients with precancerous adenoma, and healthy individuals.^108^ The results showed that fecal samples from cancer patients were significantly different from samples from healthy individuals in that an abnormal increase in common oral bacteria clostridium difficile or porphyria was observed. A similar study was subsequently carried out by Professor Peer Bork's team at the European Molecular Biology Laboratory. They sequenced the microbiomes of 156 fecal samples, some of which were from colon cancer patients, and found that the relative concentration of 22 types of bacteria predicted cancer.^109^ Bork's team found that microbial composition predicted CRC risk with 50% accuracy. When combined with blood tests, the method improved diagnostic accuracy to 70%. However, it remains unclear whether changes in colon cancer patients’ microbiomes predict disease progression.^110^

Several factors have been identified as significant and distinctive in CRC's development, including the presence of specific microbiome strains. Bacteria that are abundant in the early stages through the metastatic stages of CRC include *Fusobacterium nucleatum* and *Solobacterium moorei*. Several bacteria, such as *Atopobium parvulum* and *Actinomyces odontolyticus*, have been found to be in abundance only in adenomas and intramucosal carcinomas.^111^ Researchers examined the DNA of samples taken from CRC patients and found that *Atopobium*, *Porphyromonas Bacteroides*, and *Fusobacterium* were abundant in the samples. There are four bacteria that are associated with CRC: *Bacteroides fragilis, Escherichia coli*, *Enterococcus faecalis*, and *Streptococcus gallolyticus*. CRC patients’ fecal and tumor samples contained higher number of *F. nucleatum*, *Parvimonas*, *Peptostreptococcus*, *Porphyromonas*, and *Prevotella strains*.^112^

There are different ways in which bacteria can cause cancer. *F. nucleatum* stimulates CRC growth by provoking signaling by Toll‐like receptor 2 (TLR2)/Toll‐like receptor 4 (TLR4) and inhibiting apoptosiss.^113^ *Peptostreptococcus* contributes to the development of cancer by producing metabolites that produce an acidic and hypoxic tumor microenvironment and increase microbiome colonization. Several types of bacteria, such as *E. coli*, are genotoxic.^112^ Oncometabolites are a collection of metabolites produced by the gut microbiome and by human cancers. Several metabolites, including L‐2‐hydroxyglutarate, succinate, fumarate, D‐2‐hydroxyglutarate, and lactate, accumulate in cancer cells. Interestingly, lactic acid serves as fuel for cancer cells and contributes to cancer progression, whereas butyrate suppresses proinflammatory genes and tumor growth.^112^, ^114^, ^115^

This suggests that microbes in the body contribute to cancer development. At the very least, these studies suggest that microbial composition does influence CRC. Schloss speculated that inflammation caused by gut microbiomes creates a favorable environment for tumor formation and development. Upsetting the balance of the microbiome can result in the release of reactive oxygen species (ROS) that damage cells and their genetic material.^116^ In addition, inflammation increases the release of growth factors and angiogenic factors, which may accelerate the spread of cancer.

### 2.2 The skin microbiomes and skin cancer

Thousands of microorganisms live on the skin including bacteria, fungi, and viruses.^117^, ^118^ Like microbiomes in the gut, skin microbiomes also play important roles in resisting the invasion of harmful substances. As the largest organ in the body, the skin hosts many beneficial microorganisms, forming a physical barrier against pathogens. Recent studies have found that skin microbial dysbiosis leads to chronic inflammation, immune escape, and skin cancer.^119^, ^120^

In a tumor biopsy study, researchers used swab samples and 16S rRNA gene sequencing and found that the growth rate of *S. aureus* in the skin of squamous cell carcinoma patients was higher than in healthy individuals, and the increase in *S. aureus* was closely related to skin keratosis (precancerous lesions of squamous cell carcinoma).^121^ Malignant melanoma (MM) is the most malignant skin tumor, accounting for about 75% of all skin cancer‐related deaths.^122^ The skin microbiome in animal models of melanoma showed differences in significance between microbe composition and microbial diversity when compared with normal skin.^123^ *Fusobacterium* and *Eucoccus (Trueperella)* were found to be increased in melanoma skin samples.^123^ However, the regulatory effect of the skin microbiome on the oncogenic pathways that induce mutations remains unclear, and there is still much to learn about the role of microbiota in skin cancer.

### 2.3 Oral microbiomes and oral cancer

The association between oral microbiomes and distant cancers falls into two categories. First, microbiomes do not directly participate in the pathogenesis of cancer. However, microbial changes and carcinogenesis are correlated, and therefore microbial changes can be used as markers of cancer.^124^ Second, microorganisms are directly connected to tumorigenesis .^125^ Significantly higher levels of *Peptostreptococcus*, *Peptococcus, Fusobacterium*, *Prevotella* (especially *P. melaninogenica*), *Porphyromonas*, *Veillonella* (mainly *Veillonella parvula*), *Haemophilus*, *Capnocytophaga, Porphyromonas gingivalis, Rothia*, and *Streptococcus* have been detected in oral squamous cell carcinoma (OSCC) samples.^126^, ^127^

### 2.4 The esophagus microbiomes and esophagus cancer

The microbial composition of the esophagus is relatively stable, consisting mainly of F*irmicutes, Bacteroidetes, Actinobacteria, Proteobacteria, Clostridium*, and *Saccharibacteria*.^128^ Esophageal microorganisms are mainly classified into two types. The type I microbiome is composed of Gram‐positive bacteria and is closely associated with the normal esophagus. Type I is dominated by *Firmicutes*, and *Streptococcus* is the most dominant genus with a high relative abundance. The type II community is enriched with Gram‐negative bacteria and is mainly associated with esophageal abnormalities. Many of the bacteria in the type II microbiome are associated with the onset of esophageal cancer such as *Chaveamella, Prevotella, Haemophilus, Neisseria, Granule bacteria*, and *Fusobacterium*.^129^

Studies have shown that when esophagitis or esophageal cancer occurs, the microbial diversity of the patient's esophagus changes significantly, and the abundance of streptococcal species decreases. Gram‐negative anaerobic bacteria or microaerophiles such as *Peperamella, Prevotella, Fusobacterium*, and *Neisseria* are increased in esophageal cancer.^130^ This suggests that the Gram‐negative anaerobic microbiome may be associated with abnormal disease status and that adjusting the proportion of esophageal Gram‐positive and Gram‐negative bacteria may reduce the incidence of esophageal disease. During esophageal epithelial injury, the esophageal‐mucosal barrier is disrupted, and the bacteria translocate, affecting the esophageal microenvironment and immune homeostasis. Compared with Barrett's esophagus (BE), patients with esophageal adenocarcinoma exhibited a reduced intraesophageal pH and increases in fermented Bacillus and Lactobacillus.^131^ The abundance of *Streptococcus pneumoniae* was shown to be high in rat animal models of BE and esophageal adenocarcinoma.^132^ There is a strong association between obesity and BE and esophageal adenocarcinoma. Abdominal adipose tissue and its secretion of proinflammatory cytokines are recognized risk factors for BE and esophageal adenocarcinoma. Obese patients are also known to experience gut microbiome changes. These changes induce inflammation and change the proportion of Streptococcus and Prevotella in the upper digestive tract, which may also contribute to the development of BE and esophageal adenocarcinoma.^132^, ^133^

### 2.5 The lungs microbiomes and lung cancer

Lung cancer is a complex disease caused by interactions between the host and environmental factors.^134^ Treatment for lung cancer includes surgery, chemotherapy, radiotherapy, targeted therapy, and immunotherapy.^135^ The early diagnosis rate of lung cancer is low; and most lung cancer patients are diagnosed in the advanced stages, which have a high mortality rate. Microorganisms can maintain a microecological balance and regulate host immune responses, both of which are crucial for responding to different environmental factors. Although healthy lung tissue is considered a sterile environment, the development of high‐throughput technology has identified microbes in the microenvironment of healthy lung tissue including *Bacteroides, Firmicutes, Proteobacteria, Streptococcus, Pseudomonas, Periconella*, and *Prevotella*, where they maintain a balance between health and morbidity.^136^

Interestingly, 16s RNA sequencing of lung tissue showed that the abundance of *Bacteriaceae*, *Trichospiraceae*, and *Ruminococcaceae* in lung tissue was closely related to lung cancer risk, recurrence‐free survival, and disease‐free survival in lung cancer patients. However, the molecular mechanisms of saliva, sputum, and fecal microbes underlying the occurrence and prevention of lung cancer are not yet understood.^137^ Currently, the understanding of the lung microbiome and its impact on lung cancer and treatment is still in its infancy. Epidemiological survey data indicate that *Mycobacterium tuberculosis* (TB) is associated with lung cancer development.^138^ Compared with healthy subjects or other lung diseases, lung cancer patients have a lower alpha diversity than beta diversity.^139^ Studies have found that many gram‐negative bacteria such as *H. influenzae*, *Enterobacter*, and *E. coli* are colonized in the airways of lung cancer patients.^140^ *Peericonella, Neisseria*, and *Selenomonas* have been found in the sputum of patients with lung squamous cell carcinoma and lung adenocarcinoma carcinoma.^141^

### 2.6 The urogenital tract microbiomes and urogenital tract cancer

Although most bacteria are concentrated in the gastrointestinal tract, other parts of the body such as the genitourinary tract contain unique microbiomes.^142^ Molecular biology and cell and organ culture techniques have shown that many tissues traditionally considered sterile (including the bladder, prostate, uterus, fallopian tubes, and ovaries) harbor microbial communities when in a disease state.^143^ Anatomically, the female genital tract can be separated into the lower reproductive tract (vagina and cervix) and the upper reproductive tract (uterus, fallopian tubes, and ovaries), each with its site‐specific microenvironment. Most of the bacteria in the female reproductive tract are in the vagina, with low microbial diversity in healthy women. Lactobacillus (*L. Chris, L. garnei, L. James*, and *L. vaginalis)* is the dominant flora in the female lower reproductive tract (mainly in women in sub‐Saharan Africa).^144^ The increased consumption of lactic acid bacteria was found to be involved in a variety of female diseases such as sexually transmitted diseases, preterm birth, spontaneous abortion, and pelvic inflammation.^145^ Owing to the presence of lactic acid, the microenvironment of the vagina remains at pH < 4.5, which is the main mechanism for the self‐cleaning of the vagina. Studies have shown that vaginal flora disorders are closely associated with gynecological tumors. For example, it is well established that papillomaviruses (HPV) infection and cervical cancer occurrence are closely related.^144^ A recent study found that during HPV infection, the microenvironment of the cervix changes, and HPV cooperatively promotes tumorigenesis.^146^ Clinical studies showed that vaginal lactobacillus depletion and overgrowth of anaerobic bacteria were positively associated with an increased risk of cervical cancer. Lactobacillus predominance and a significant increase in vaginal microbiome diversity were found in patients with cervical precancerous lesions and cancer, compared with healthy women.^147^ In patients at high risk of cervical cancer, local cervicovaginal conditions such as increased proinflammatory cytokines and cancer‐specific biomarkers, vaginal pH abnormalities, and excessive depletion of lactic acid bacteria can directly or indirectly lead to cervical cancer.

Endometrial cancer is the most common gynecological cancer in developed countries. Several factors including obesity, inflammation, metabolic imbalance, and postmenopausal estrogen therapy are major risk factors for the type I endometrial cancer.^148^ These factors are associated with changes in the gut and vaginal microbiome.^117^, ^149^ The close association between the gut microbiome, estrogen metabolism, and obesity suggests that the microbiome may contribute in the etiology of endometrial cancer.^117^, ^119^ Estrogen compounds can influence the vaginal microbial community, indicating that regulating estrogen can affect the vaginal microbiome and therefore influence endometrial hyperplasia and cancer development.^149^ A study using a 3D human vaginal epithelial model showed that *A. vaginae* induced the production of proinflammatory cytokines and antimicrobial peptides. *Antibiotics*, *porphyrin*, *dialysis bacteria*, *Ruminococcus*, *anaerobic bacteria*, *Treponema*, *Bacteroides*, and *Spirospira* were found to be significantly enriched in tumors. *Proteobacteria*, *Porphyromonas*, and an abnormal vaginal pH (>4.5) also occur in patients with endometrial cancer.^150^

In addition, cancer of the ovary is one of the deadliest malignancies in women. Dysbiosis of the genital microbiome has been implicated in ovarian cancer development and has been suggested as a potential biomarker of the disease. Clinical data suggest a unique, potentially pathogenic profile in the ovarian microbiome among ovarian cancer patients, with organisms such as *Brucella, Mycoplasma*, and *Chlamydia*.^151^ Moreover, *Proteobacteria*, especially *Acinetobacter* are increased in the ovarian microbiome. Although preliminary studies have demonstrated that the presence of various bacteria in ovarian cancer tissues is associated with ovarian inflammation and that the highly hypoxic tumor microenvironment favors the recruitment and growth of anaerobic microorganisms, the causal relationship between the microbiome and ovarian cancer remains unclear.^152^ Studying tissue specificity of the ovarian cancer microbiome and determining its role in tumors could aid in understanding the association between the female reproductive tract microbiome and ovarian cancer.

Traditionally, urine is thought to be sterile. However, recent studies have found that microorganisms are present in the urinary tract and bladder, and that the urinary microbiome may change with age. The female urinary microbiome contains mainly *Lactobacillus* and *Gardnerella*, while the male microbiome is primarily *Corynebacterium, Staphylococcus*, and *Streptococcus*. Microorganisms in the urethra are strongly associated with cancers in the genitourinary system.^153^ Most current studies focus only on bacteria in the urogenital tract, with less attention to other microbes such as *fungi* and *viruses*. Many infectious‐related cytokines lead to chronic inflammation, which, in turn, drives cellular carcinogenesis. Microorganisms in the urinary tract can control the pathogenic bacteria growth and inhibit tumorigenesis, but the role of the urogenital microbiome in regulating pathogenic infections and mediating cancer development requires further clarification.

It is not yet clear whether the urinary microbiome affects bladder cancer progression, or whether the composition, diversity, or abundance of microorganisms is associated with bladder cancer. A high abundance of *Fusobacterium, Actinomyces, Facracter*, and *Campylobacter* was found in the urine of patients with bladder cancer, while *Streptococcus* and *Corynebacterium* were higher in the urine of healthy people. The abundance of bacteria was increased in patients with bladder cancer compared with urine from nontumor patients, but there was no difference in diversity.^154^ Studies of prostate cancer suggest that the urinary microbiome in patients with prostate cancer may contain proinflammatory bacteria, and the chronic proliferative inflammation caused by these proinflammatory bacteria may be a risk factor for the development of prostate cancer. However, the association between prostatitis and prostate cancer and the urinary microbiome remains unclear.^155^, ^156^

As research on genitourinary microorganisms and genitourinary cancers is in the early stages, data from large populations are needed to reveal the relationship between genitourinary microorganisms and cancer. Analysis of the role of genitourinary microorganisms in tumorigenesis is promising for the treatment of cancer.

### 2.7 The blood and lymphatic system microbiomes and cancer

Currently, studies on leukemia and microbiomes have primarily focused on the relationship between childhood leukemia and gut microbes. Acute leukemia is the most common cancer in children and the most common cause of cancer‐related deaths in childhood. It was found that patients without infectious complications had an increased relative abundance of *Bacteroidetes* and *Proteus faecacterium*, while patients with concurrent infections had an increased abundance of aerobic bacteria.^157^ Because chemotherapy damages the intestinal mucosal barrier, bacteria translocate into the blood, which can lead to diarrhea, abdominal pain, low diversity of enterococcus and porphyraceae, and increased abundance of C reactive protein.^158^ Microorganisms mainly secrete SCFAs such as butyrate. Butyrate has a key nutritional effect on the intestinal epithelial barrier, and when chemotherapeutic drugs destroy the intestinal epithelial barrier, the microbial abundance of the Trichospiraceae family decreases, aggravating microbial infection.^159^ The genetic predisposition for leukemia is also closely related to commensal microorganisms.^160^ Bone marrow suppression and immunosuppression are common in children with leukemia. One study found that after the induction and reinduction of patients, microbiome diversity in children's feces with leukemia was decreased significant, and the proteobacteria including Enterobacteriaceae and Pseudomonas were also reduced. Analysis of the changes in intestinal microbial diversity and composition before and after treatment can be used to monitor the occurrence of adverse chemotherapy reactions.^161^

Although recent studies have explored the relationship between the gut microbiome and acute childhood leukemia, the relationship between human T cell leukemia virus type 1 (HTLV‐1)‐related adult T cell leukemia (ATL) and intestinal microbes is rarely reported. The prognosis of ATL is very poor; the median survival of acute ATL is 8 months, the 4‐year overall survival is 11%, and the treatment options are very limited. The FBXW7 proteins in the F‐box protein family were found to play a role in tumor protein phosphorylation‐dependent ubiquitination and proteasomal degradation.^162^ The absence of FBXW7 could serve as an independent prognostic marker and was found to be significantly associated with tumor cell resistance to chemotherapeutic agents and poor disease prognosis.^163^, ^164^, ^165^ It is a gut commensal anaerobic bacterium that produces large amounts of thioredoxin and nitroreductase, reduces oxidative stress associated with CYB5RL mutations, and inhibits cell death. However, it is unclear whether FBXW7 is involved in leukemogenesis.^166^, ^167^ Notch1 signaling activation promotes proliferation in adult acute leukemia cells and regulates hepatic glucose production and lipid synthesis. It has been shown that inhibiting Notch1 signaling in HepG2 cells increased the expression of fatty acid oxidation genes and fatty acid oxidation rate.^168^ In adult acute leukemia, JAG1 overexpression contributed to the Notch1 signaling pathway and the migration of HTLV‐1 ‐transformed ATL cells.^169^

This suggests that gut microbes may be involved in ATL development through the SCFA pathway. Because the gut microbiome develops with age, further confirmation of whether the fecal microbiome can provide information on the entire gut microbiome is needed. Fasting and breastfeeding can both modulate the gut microbiome to reverse the progression of childhood leukemia. However, the specific microorganisms that promote or reverse leukemogenesis and progression remain poorly defined. Therefore, the identification of specific microorganisms closely related to leukemogenesis and progression, and elucidation of gut microbiome mechanisms promotes or reverses leukemia is imperative in providing new strategies for the treatment of leukemia.

Very few studies have focused on lymphoid tissue and microorganisms. In a study on the relationship between changes in human lymphatic structure/intestinal function and gut microbes, researchers determined that the abundance of *Prevotella* and *Bacteroidetes‐Prevotella‐porphyromona* (BPP) increased in the gut microbiome. This was the first human study to link changes in lymphatic structure/intestinal function and the gut microbiome.^170^ Subsequent studies found that the incidence of *H. pylori*‐negative mucosa‐associated lymphatic tissue (MALT) lymphoma was related to the presence of *H. pylori* in some patients. Interestingly, alpha diversity was significantly reduced in *H. pylori*‐negative MALT lymphoma patients compared to controls (*p* = 0.04). In addition, *Burkholderia* and *Sphingomonas* were significantly increased in MALT lymphoma patients, but *Prevotella* and *Pellona* were lower, suggesting that an altered microbiome may be involved in the pathogenesis of *H. pylori*‐negative MALT lymphoma.^171^ Patients with gastrointestinal follicular lymphoma (GI‐FL) had significantly less alpha diversity compared to controls. The microbial composition between the two groups was significantly different, and the content of sporophyte, *Rhodella*, *Prevotella*, and *Doupecaceae* was significantly lower in GI‐FL patients, suggesting that this microbiome may be play a role in the pathogenesis of GI‐FL.^172^

### 2.8 Others

#### 2.8.1 The hepatitis viruses and liver cancer

Hepatitis B and C viruses play a role in the pathogenesis of primary hepatocellular carcinoma (HCC).^173^ By integrating viral genes and mutating viral proteins (mostly HBx and PreS), HBV can induce unstable insertion and deletion of host genes.^174^ Genotype C and D of chronic hepatitis B patients with a promoter A1762T/G1764A mutation are at higher risk of HCC than those with genotype A and B.^175^ DNA integration of HBV occurs more frequently in tumor tissues compared with adjacent normal liver tissues. Approximately 40% of HBV gene break points located in the core area of virus enhancer.^176^ HBV DNA integrated into the host genome and caused genetic damage and chromosomal instability. It has a selective advantage for tumor progression. Viral proteins (HBx, HBc, and PreS) mutants can affect cell function, activate oncogenic pathways and increase the sensitivity of hepatocytes to these mutants. HBx viral protein play a role in carcinogenesis by inducing the cytoplasmic isolation of chromosomal maintenance protein 1 (Crm1) and promoting nuclear factor kappa‐B (NF‐κB) enter into the nucleus. On the other hand, HBx viral protein can also promote carcinogenesis by maintaining centrosome integrity though Crm 1 and affecting mitosis checkpoint. Telomerase activation is observed in over 90% of HCC patients and is closely related to HCC.^177^ C‐terminally truncated middle surface protein of hepatitis B virus (MHBst) and wild‐type/truncated HBx proteins activate the transcripts promoter of TERT in HBV‐associated HCC.^178^ Many signaling pathways were activated in HCC such as Wnt/β‐Catenin, cell cycle, oxidative stress metabolic pathway, Ras/ MAPK, and so on.^179^

Whether HCV can induce HCC is unknown, but it is a primary risk factor for the disease. It depends on the interaction among virus, host and environmental factors when HCV carriers developed into HCC. The HCV genome cannot be integrated into the host genome, but it can damage the host liver cells DNA through viral proteins and ROS producing by some certain carcinogenesis pathways, causing gene mutation and HCC occurrence.^180^ It is possible for HBV carriers or HCV carriers to develop cancer due to some certain factors, such as sex, age, family history, viral load, infection time, coinfection with other virus (HDV, HIV, HBV/HDV), environmental factors, and so on.^181^ The intestinal microbiomes also might be involved in the development of HCC.

#### 2.8.2 The HPV and cervical cancer

Human papillomavirus is the most common sexually transmitted virus in the world.^182^ In addition, more than 99% of cervical cancer cases are caused by infection with high‐risk HPVs (hrHPVs).^183^ Since 85–90% of hrHPVs infections are transient and can be spontaneously cleared by the host's immune system, HPVs infection alone cannot cause the disease. There is only a 10–15% chance that cervical intraepithelial neoplasia will progress to invasive cervical cancer with persistent infections.^184^

Additionally to their transformational properties, HPV‐infected cells actively influence the local microenvironment. As a result of creating a supportive postinfection microenvironment (PIM), virus‐infected cells play an important role in cervical cancer development.^185^, ^186^ A PIM is formed by interactions between virus‐infected cells, immune cells, and host stroma, as well as their derived components (chemokines, cytokines, extracellular vesicles, and metabolites).^187^ As a result, PIM actively alters the local microenvironment, thereby promoting the persistence and spread of the virus and the development and progression of cervical cancer. Understanding PIM properties and its role in HPV infection and disease progression can help identify potential biomarkers to improve diagnosis and prognosis.

#### 2.8.3 The EBV and nasopharyngeal carcinoma

In humans, EBV is the first virus that causes cancer. There are several diseases that can be caused by or associated with EBV infection, including infectious mononucleosis, burkitt lymphoma, and nasopharyngeal carcinoma (NPC).^188^ A NPC is a rare epithelial cancer of the nose and throat, divided into three pathological subtypes (keratinized squamous carcinoma, nonkeratinized squamous carcinoma, and basic squamous carcinoma).^189^ NPC is almost always caused by EBV infection, which is the most common pathogenic factor.^190^ When EBV is infected into B lymphocytes, it will remain latent at the cytoplasm for a long time. As a result, EBV latent infection is a characteristic of precancerous nasopharyngeal epithelium. Infected epithelial cells express viral genes such as EBER, EBNA1, LMP1, and LMP2A, which can initiate nasopharyngeal epithelial tumor growth.^191^ Additionally, it is not fully understood how EBV infection contributes to NPC development and progression. According to recent genomic analyses, the NF‐κB signaling pathway, caused by overexpression of the EBV viral oncoprotein, may play a role in NPC.^192^

## 3 MECHANISMS BY WHICH HUMAN CARCINOGENIC MICROBIOMES PROMOTE CANCER

Carcinogenic microbiomes can suppress human immune system directly, active immunosuppressive factors in tumor microenvironment, inhibit immune cells activity in tumor microenvironment, induce cell mutation, and promote cell proliferation. We reviewed the mechanism of human carcinogenic microbiomes promoting cancer in this section.

### 3.1 Alteration in immune system activity

Gut microbiomes not only participate in the pathogenesis of CRC but also secrete inflammatory factors and destroy the integrity of the intestinal barrier,^193^ activating cellular metabolic pathways and promoting breast cancer,^131^, ^194^ and HCC.^132^, ^195^ One study using a tumor‐bearing mouse model showed that *H. pylori* and interleukin‐22 (IL‐22) upregulated the expression of matrix metalloproteinase 10 (MMP10) in gastric epithelial cells through the extracellular signal‐regulated kinase (ERK) pathway, producing chemokine ligand 16 (CXCL16).^196^, ^197^ This recruited CD8+ T cells and induced an inflammatory response, which damaged the gastric mucosa and promoted gastric carcinogenesis .^198^ In a mouse model of CRC, enterotoxin *Bacteroides fragilis* (ETBF) stimulated the immune response through T helper 17 cells (Th17) and promoted colon tumor growth.^137^, ^138^, ^199^ Similarly, *E. coli* expressing the genomic island polyketide synthase (pks+) enhanced tumorigenesis in preclinical CRC models and were found to be enriched in human CRC tissues .^200^, ^201^ Furthermore, pks + *E. coli* produces the genotoxin colibactin, which alkylates DNA, resulting in the formation of DNA adducts (a form of potentially mutagenic DNA damage) in colonic epithelial cells. The recent biochemical resolution of this process illustrates how host‐microbe interactions may lead to cancer.^202^ *Streptococcus gastrop* (*Peptostreptococcus anaerobius*) interacts with TLR2 and TLR4 in colon cells to regulate a variety of immune cell types including myeloid‐derived suppressor cells (MDSCs), tumor‐associated macrophages (TAMs), and granulocyte tumor‐related neutrophils, thus promoting colon carcinogenesis.^203^ *F. nucleatum* is associated with various types of cancer including CRC.^204^ *F. nucleatum* was shown to bind to epithelial cadherin (E‐cadherin) through FadA and Fap2, inducing β‐Catenin signaling, regulating the inflammatory and carcinogenic response, and promoting tumorigenesis.^123^, ^205^ *P. anaerobius* has been shown to bind to tumor cell surface integrins via a cell surface protein, putative cell wall binding repeat 2 (PCWBR2), activating the PI3K–AKT pathway and the NF‐κB cascade and inducing an inflammatory response that promoted tumor cell proliferation.^203^ *P. anaerobius* recruited tumor‐infiltrating, promoted cell proliferation, and triggered an inflammatory response in the tumor microenvironment through the PI3K/AKT/NF‐kB signaling pathway.^129^

**FIGURE 2:**
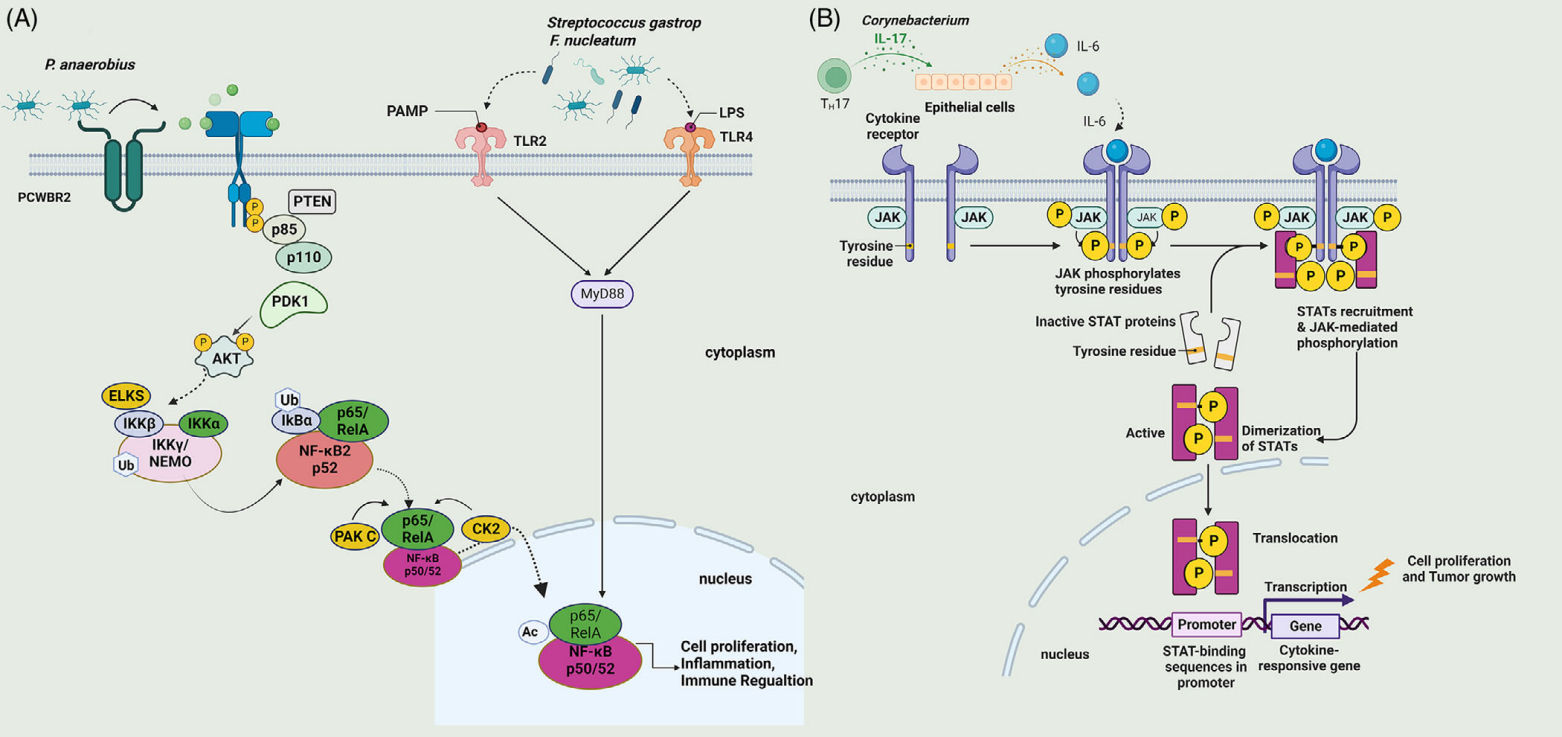
Alteration of immune system activity. (A) *P. anaerobius* recruited tumor‐infiltrating, promoted cell proliferation, and triggered the inflammatory response in the tumor microenvironment through the PI3K/AKT/NF‐kB signaling pathway. *F. nucleatum* induced lymphocyte death through the TLR4/MYD88 pathway, promoting tumor development. (B) *Corynebacterium* induced IL‐17 production, upregulated IL‐6, and activated the JAK/STAT3 signaling pathway to induce cell proliferation and tumor growth.

Various studies have demonstrated several different mechanisms in OSCC such as the promotion of the production of inflammatory cytokines as well as cellular proliferation and invasion by *P. gingivalis* and *F. nucleatum*.^206^ *F. nucleatum* interacted with T‐cell immunoglobulin and ITIM domain (TIGIT) to inhibit NK cell cytotoxicity, which induced lymphocyte death through the TLR4/MYD88 pathway and promoted tumor development and induced resistance to chemotherapy^207^ (Figure 2A). *F. nucleatum* stimulated cell proliferation and induced increased levels of IL‐2 and MMPs necessary for tumor invasion and metastasis.^208^ Oncogene transcription activity increased greatly due to these changes, as well as levels of proinflammatory cytokines.^208^ *Fusobacterium* may promote tumor proliferation through Fap2, T cell immunoglobulin, and TIGIT, and inhibit the cytotoxicity of NK cells.^209^, ^210^ Skin swabs from patients with acral melanin showed a high abundance of the *Corynebacterium* genus in stage III/IV MM patients compared with stage I/II patients. *Corynebacterium* induced IL‐17 production, upregulated IL‐6, and activated STAT3 signaling, which induced melanoma growth (Figure 2B). This indicates that *Corynebacterium* can promote the development of MM through an IL‐17‐dependent pathway. Intratumoral injection of *Corynebacterium* acnes induced Th1 cytokines such as IL‐12, tumor necrosis factor (TNF), and interferon (IFN), and inhibited the growth of melanoma cells.^211^, ^212^

Studies have shown that *Fusobacterium* upregulated cervical IL‐4 and transforming growth factor 1 expression and inhibited the vaginal immune microenvironment.^133^ In the vaginal microenvironment of cervical cancer patients, proinflammatory factors such as IL‐36, chemotaxis factors such as IFN‐induced protein 10 (IP10), macrophage inflammatory protein 1 (MIP‐1β), and RANTES, hematopoiesis factors such as FLT3 ligand, and adaptive immune response cytokines such as IL‐2, IL‐4, and soluble CD40 ligand were associated with increased nonlactobacilli microbial growth.^133^ *S. gastrop* (*P. anaerobius*) has been shown to interact with TLR2 and TLR4 on colon cells to regulate a variety of immune cells including MDSCs, TAMs, and granulocyte tumor‐related neutrophils, thus promoting colon carcinogenesis.^203^ Additionally, the specific chemokine genes that are upregulated in ESCC tissues with *F. nucleatum* suggest that *F. nucleatum* might contribute to the growth of esophageal tumors by activating chemokines such as CCL20.^213^

Studies have confirmed that the secretion of *S. epidermidis* activated regulatory T cells and inhibited inflammatory responses in the skin. Both animal and cell experiments have confirmed that *S. epidermidis* produces 6‐n‐hydroxyaminophosphorus (6HAP), which inhibits DNA synthesis and UV‐induced tumor growth.^214^, ^215^ Furthermore, it was also reported that *S. epidermidis* and its derived LTA upregulated TRAF1, CASP14, and CASP5 and improved the survival of melanoma cells.^216^ *C. acnes* promoted apoptosis, enhanced fecal porphyrin secretion, upregulated TNFα, and promoted the death of residual melanoma cells after radiotherapy.^217^ Inhibition of *S. aureus* infection with topical mupirocin and the oral antibiotic dicloxacillin also improved clinical symptoms of CTCL, which was likely linked to an increase in IL‐2 and STAT3 signaling activation.^218^

Skin microbial dysbiosis, activation of the skin immune system, production of microbial metabolites and toxins, destruction of barriers and UV radiation may all play a role in skin cancer occurrence and development.^219^ However, the regulatory effect of the skin microbiome on the oncogenic pathways that induce mutations is not clear, and further studies are needed to elucidate the role of the skin microbiome in skin cancer.

Cancer‐causing bacteria are largely induced by inflammation‐associated cytokines.^208^ Leakage of microbial products from the intestinal lumen to the peripheral circulation increases the levels of lipopolysaccharide (LPS) in the peripheral circulation and activates chronic inflammation, leading to the development of non‐Hodgkin's lymphoma (AIDS‐NHL) associated with acquired immunodeficiency syndrome (AIDS).^220^ Microorganisms can regulate the immune response, alter epigenetic markers of cells, modify target cells, activate B lymphocytes, and cause patients with Sjogren's syndrome (SS) to develop B cell lymphoma.^221^, ^222^, ^223^ *S. aureus* is closely associated with cutaneous T‐cell lymphoma and promotes the progression of this disease.^224^ Staphylococcal‐toxin (a‐hemolysin) inhibits cytotoxic responses by T cells and lead to immune escape.^225^ Staphylococcal enterotoxin produced by *S. aureus* can activate the STAT5 protein, causing an increase in Th2 cells.^226^ Further, Staphylococcal enterotoxin interferes with the removal of malignant tumor cells by cytotoxic T cells, inhibiting autoimmunity and increasing tumor immune tolerance.^225^

Epithelial biopsy specimens of BE are enriched in proinflammatory cytokines, especially IL‐1b.^24^ Moreover, a number of inflammatory factors are released under inflammatory conditions, such as IL‐6 and IL‐23, which promote the immune response between microorganisms and hosts by activating the inflammatory response, which may ultimately lead to cancer.^227^ The esophageal type II microflora can produce abundant LPS due to the presence of Gram‐negative bacteria.^207^, ^228^ In addition to affecting cyclooxygenase 1/2, LPS can directly affect the lower esophageal sphincter, resulting in an increase in intragastric pressure and promoting the GERD occurrence, which leads to EAC development.^207^, ^229^ There is interesting evidence that in patients with BE and EAC, TLR4 is being expressed more strongly as a natural ligand of LPS.^207^ It is thought that TLR4 receptor activation triggers the NF‐B pathway, which contributes to inflammation‐related carcinogenesis,^230^, ^231^ and is implicated in early BE.^232^ Thus, inflammation and malignant transformation of the esophagus can be triggered by activating LPS‐TLR4‐NF‐kB.^207^, ^233^ As well, After BE cells were treated with LPS, Nadatani et al. observed increased expression of NOD‐like receptor protein 3, caspase‐1 activity, and secretion of IL‐1b and IL‐18. They hypothesized that LPS activates ROS, thereby promoting cancer development.^121^, ^234^ Recent studies have found that the host‐lung microbiome interaction is closely related to lung cancer development. An animal model of lung cancer using K‐ras/p53‐mutant (KP) mice showed a decreased incidence of lung cancer in germ‐free mice compared with SPF mice. Reduced airway microbial microbe diversity was found in SPF tumor‐bearing mice. In germ‐free mice, expression of IL‐1, IL‐23, IL‐17, and ROR‐γt were decreased in γδ T cells from peripheral lymph nodes and the spleen.^235^ Treatment with aerosolized antibiotics in tumor‐bearing mice revealed a reduced number of regulatory T cells (Tregs), enhanced activation of immune effector cells, increased immune surveillance function in the lungs, and suppressed tumor metastasis, which was correlated with decreased enrichment of Streptococcus, Proteobacteria, and Actinobacteria in lung tissue.^149^, ^235^ These studies suggest that the lung microbiome has a direct link to immune regulation, but exactly how this microbiome affects immune cells in the tumor microenvironment is currently unknown.

### 3.2 Influence on host cell proliferation and death

In a mouse CRC model, ETBF stimulated the immune response via T helper 17 cells (Th17) and promoted colon tumor growth.^123^, ^236^ Additionally, a preclinical study found that *E. coli* expressing genetically altered polyketide synthase (pks+) enhanced tumor formation in mice. pks+ expression was found to be significantly enriched in human cancer tissues.^200^ DNA adducts (potentially mutagenic DNA damage) are created when *pks+ E. coli* produce the genotoxin colibactin. This process was demonstrated via the recent biochemical resolution of this interaction as a possible cause of cancer.^202^ *F. nucleatum* is associated with various types of cancer, including CRC. *F. nucleatum* was shown to bind to epithelial cadherin (E‐cadherin) through FadA and Fap2, inducing β‐Catenin signaling and regulating the inflammatory and carcinogenic responses, ultimately promoting tumorigenesis.^123^ *P*. *gingivalis* and *F. nucleatum* have been shown to induce the production of inflammatory cytokines, cell proliferation, and cellular invasion in OSCC through a variety of different mechanisms.^206^ *P. gingivalis* stimulated the formation of IL, TNF‐α, and MMP while inhibiting apoptosis.^206^, ^237^

There is no doubt that chemokines, along with their receptors, play an important role in the development and progression of cancer.^238^, ^239^, ^240^ Cancer cells were empowered by CCL20 stimulation in vitro, according to Wang et al.^241^ EC may be caused by genetic toxins or cancer‐promoting metabolites produced from bacteria, as well as microbiome components. To illustrate, by secreting swelling toxins, Gram‐negative bacteria may damage host DNA,^242^, ^243^, ^244^ and subsequent repair of that damage may cause EC.^245^ Gabriel et al. found that mammalian epithelial cells exposed to *E. coli* exhibited DNA damage responses, followed by cell division with signs of incomplete DNA repair, resulting in anaphase bridges and chromosome aberrations. A significant increase in DNA mutation frequency was observed among cells exposed to *E. coli*, as well as the formation of anchorage‐independent colonies, demonstrating that E. coli infection has the potential to be mutagenic and transformative.^243^

**FIGURE 3:**
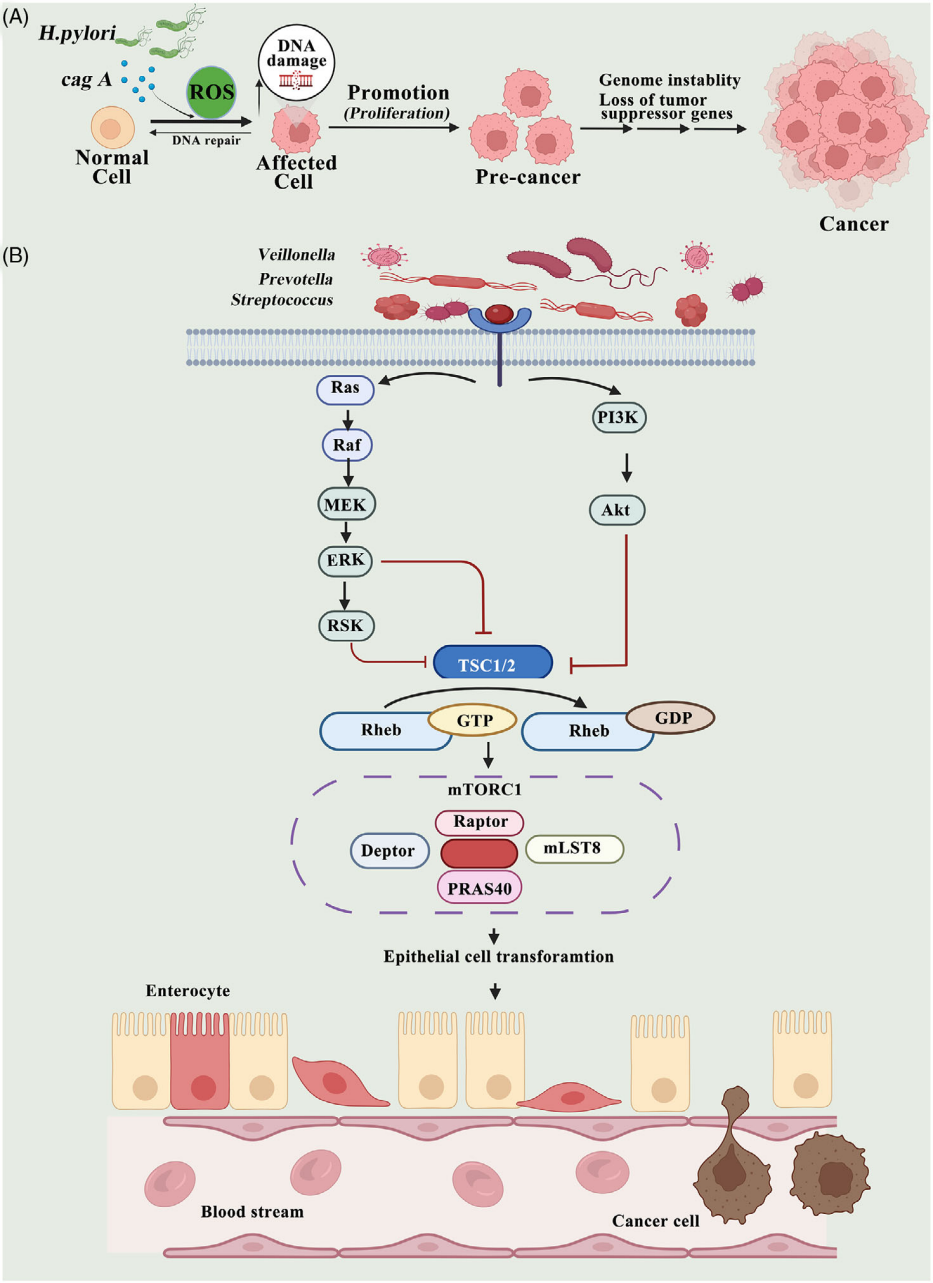
Influence on host cell proliferation and death. (A) Cytotoxin‐associated gene A (CagA) produced by *H. pylori* can stimulate inflammation and lead to the development of cancer. CagA induces DNA damage through host‐mediated upregulation of the production of reactive oxygen species in the cytoplasm and promotion of the DNA damage response. (B) The connection between microbial components and lung cancer can be explained by the production of metabolites. *Veillonella*, *Prevotella*, and *Streptococcus* bacteria induce epithelial cell transformation and promote epithelial cell transformation through activation of the PI3K and ERK signaling pathway.

Moreover, *H. pylori* produce cytotoxin‐associated gene A (CagA) or vacuolating cytotoxin A and can cause inflammation and cancer.^246^ By increasing the level of ROS in the body, CagA can induce DNA damage by upregulating host‐mediated ROS production.^214^, ^247^ It is also known that vacuolating cytotoxin A has a tendency to alter membrane permeability and cause an increase in apoptosis rates.^209^ The study by Li et al. demonstrated that a CagA1‐positive *H. pylori* can break the DNA in squamous epithelial cells in the esophagus, leading to atypical hyperplasia and causing ESCC carcinogenesis. Further mechanistic research found that *H. pylori* infection induces ROS production in the cytoplasm, which promotes the DNA damage response (Figure 3A).^248^

Dysbiosis of the lung microbiome may influence cancer progression by causing dysregulated immune responses, characterized by the overactivity of inflammatory cells such as M1 macrophages, Th1 cells, or γδ T cells.^249^, ^250^ Currently, it is unclear exactly how lung cancer is related to the airway microbiome and immune regulation.^251^ The connection between microbial components and lung cancer can be explained by the production of metabolites; however, this has been insufficiently researched compared to the relationship between dysbiosis and chronic inflammation. Researchers have discovered that as a result of exposure to *Veillonella*, *Prevotella*, and *Streptococcus bacteria*, epithelial cells are transformed both in vitro and in vivo by activating ERK and PI3K (Figure 3B).^131^, ^252^, ^253^

## 4 MICROBIOME TREATMENT

The gut microbiome can modulate the efficacy of anticancer drugs.^254^ Alterations in the gut microbiome are associated with tumor resistance to chemotherapeutic agents or immune checkpoint inhibitors (ICIs),^255^, ^256^ and modulation of the gut microbiome by antibiotics, probiotics, fecal microbiome transplantation (FMT), and nanotechnology may enhance the antitumor effects of chemotherapeutic agents and ICIs.^257^

In recent years, the application of precision medicine in cancer treatment has greatly improved the disease‐free survival and quality of life of cancer patients. However, treatment failure due to patient hyporesponsiveness to immunotherapy is a great challenge in cancer treatment. Owing to the complexity of the host immune response, the mechanisms of immunotherapy are not well defined.^136^, ^137^ Microorganisms can promote tumor regression by enhancing the host immune response. There are new possibilities for effective cancer treatment with the microbiome.^139^ The microbial treatment of cancer primarily includes fecal transplantation, targeted use of antibiotics, probiotics, bacteria, prebiotics, and phages.^140^, ^141^ FMT is the process of introducing feces from healthy donors (or the cryopreserved microbial content) into a patient's colon.^258^ Interestingly, the fecal enema was used to treat pseudomembranous colitis clinically and achieved better results than FMT. FMT has been approved for the treatment of recurrent *C. difficile* infections, based on *C. difficile* treatment guidelines (2013). Increasing evidence has also demonstrated the beneficial effects of FMT in the treatment of other diseases such as intractable functional constipation, inflammatory bowel disease, and hematological malignancies.^22^ In recent years, clinical trials of FMT have been carried out (NCT03812705, NCT04729322, NCT04163289, NCT03819296). For example, patients with PD‐1 refractory melanoma are being treated with FMT alone or in combination with pembrolizumab (NCT03353402). Some of the patients treated with the combination therapy had increased T lymphocyte infiltration into the tumor tissue and achieved remission of clinical symptoms. In clinical studies using FMT treatment for acute myeloid cell leukemia, multiple myeloma, myelodysplastic syndrome, and lung cancer, 40% of patients initially developed neutropenia. However, the final study results indicated that FMT therapy was safe and effective.^259^ FMT is a relatively new approach for altering gut microbiome composition, so a long‐term safety assessment is still needed (Table 2).

**TABLE 2:** Clinical trials in microbiome treatment (https://www.clinicaltrials.gov/).

	NCT number	Title	Status	Conditions	Interventions	Phase
1	NCT04857697	Effects of Probiotics on the Gut Microbiome and Immune System in Operable Stage I‐III Breast or Lung Cancer	Recruiting	Anatomic Stage I Breast Cancer AJCC v8 Anatomic Stage II Breast Cancer AJCC v8 Anatomic Stage III Breast Cancer AJCC v8 Breast Adenocarcinoma Stage I Lung Cancer AJCC v8 Stage II Lung Cancer AJCC v8 Stage III Lung Cancer AJCC v8	Procedure: Biopsy Procedure: Biospecimen Collection Drug: Probiotic Procedure: Therapeutic Conventional Surgery	Early Phase 1
2	NCT03819296	Role of Gut Microbiome and Fecal Transplant on Medication‐Induced GI Complications in Patients With Cancer	Recruiting	Clinical Stage 0 Cutaneous Melanoma AJCC v8 Clinical Stage I Cutaneous Melanoma AJCC v8 Clinical Stage IA Cutaneous Melanoma AJCC v8 Clinical Stage IB Cutaneous Melanoma AJCC v8 Clinical Stage II Cutaneous Melanoma AJCC v8 Clinical Stage IIA Cutaneous Melanoma AJCC v8 Clinical Stage IIB Cutaneous Melanoma AJCC v8 Clinical Stage IIC Cutaneous Melanoma AJCC v8 Clinical Stage III Cutaneous Melanoma AJCC v8 Clinical Stage IV Cutaneous Melanoma AJCC v8	Other: Best Practice Other: Biospecimen Collection Procedure: Endoscopic Procedure Procedure: Fecal microbiome Transplantation Biological: Infliximab Other: Laboratory Biomarker Analysis Drug: Prednisone Biological: Vedolizumab	Phase 1 Phase 2
3	NCT05020574	Microbiome and Association With Implant Infections	Recruiting	Breast Cancer Breast Cancer Female Genetic Predisposition to Disease	Drug: Cephalexin	Phase 2
4	NCT04552418	Intestinal Microbiome Modification With Resistant Starch in Patients Treated With Dual Immune Checkpoint Inhibitors	Recruiting	Solid Tumor	Drug: Potato starch	Early Phase 1
5	NCT04875728	The Impact of an Antibiotic (Cefazolin) Before Surgery on the Microbiome in Patients With Stage I‐II Melanoma	Recruiting	Clinical Stage I Cutaneous Melanoma AJCC v8 Clinical Stage IA Cutaneous Melanoma AJCC v8 Clinical Stage IB Cutaneous Melanoma AJCC v8 Clinical Stage II Cutaneous Melanoma AJCC v8 Clinical Stage IIA Cutaneous Melanoma AJCC v8 Clinical Stage IIB Cutaneous Melanoma AJCC v8 Clinical Stage IIC Cutaneous Melanoma AJCC v8 Pathologic Stage I Cutaneous Melanoma AJCC v8 Pathologic Stage IA Cutaneous Melanoma AJCC v8 Pathologic Stage IB Cutaneous Melanoma AJCC v8	Drug: Cefazolin Procedure: Resection	Phase 1
6	NCT03686202	Feasibility Study of Microbial Ecosystem Therapeutics (MET‐4) to Evaluate Effects of Fecal Microbiome in Patients on Immunotherapy	Recruiting	All Solid Tumors	Biological: MET‐4	Early Phase 1
7	NCT03959241	TAC/MTX vs. TAC/MMF/PTCY for Prevention of Graft‐ versus‐Host Disease and Microbiome and Immune Reconstitution Study (BMT CTN 1703/1801)	Active, not recruiting	Acute Leukemia Chronic Myelogenous Leukemia (CML) Myelodysplasia Lymphoma	Procedure: Mobilized Peripheral Blood Stem Cell graft with Tacrolimus/Methotrexate Drug: Tacrolimus Drug: Methotrexate Procedure: Mobilized Peripheral Blood Stem Cell graft with Tacrolimus/Mycophenolate Mofetil/ Post‐Transplant Cyclophosphamide Drug: Mycophenolate Mofetil Drug: Cyclophosphamide	Phase 3
8	NCT03817125	Melanoma Checkpoint and Gut Microbiome Alteration With Microbiome Intervention	Active, not recruiting	Metastatic Melanoma	Drug: Placebo for antibiotic Drug: Vancomycin pretreatment Drug: Nivolumab Drug: Matching Placebo for SER‐401 Drug: SER‐401	Phase 1
9	NCT03812705	Fecal microbiome Transplantation for Steroid Resistant/Dependent Acute GI GVHD	Recruiting	Hematopoietic and Lymphoid Cell Neoplasm	Biological: fecal microbiome transplantation	Phase 2
10	NCT04729322	Fecal microbiome Transplant and Re‐introduction of Anti‐PD‐1 Therapy (Pembrolizumab or Nivolumab) for the Treatment of Metastatic Colorectal Cancer in Anti‐PD‐1 Non‐Responders	Recruiting	Metastatic Colorectal Adenocarcinoma Metastatic Small Intestinal Adenocarcinoma Stage IV Colorectal Cancer AJCC v8	Procedure: Biopsy Procedure: Fecal microbiome Transplantation Drug: Fecal microbiome Transplantation Capsule	Early Phase 1
11	NCT04163289	Preventing Toxicity in Renal Cancer Patients Treated With Immunotherapy Using Fecal microbiome Transplantation	Recruiting	Renal Cell Carcinoma	Drug: Fecal microbiome Transplantation	Phase 1
12	NCT03819296	Role of Gut Microbiome and Fecal Transplant on Medication‐Induced GI Complications in Patients With Cancer	Recruiting	Clinical Stage 0 Cutaneous Melanoma AJCC v8 Clinical Stage I Cutaneous Melanoma AJCC v8 Clinical Stage IA Cutaneous Melanoma AJCC v8	Other: Best Practice Other: Biospecimen Collection Procedure: Endoscopic Procedure	Phase 1 Phase 2
13	NCT03353402	Fecal microbiome Transplantation (FMT) in Metastatic Melanoma Patients Who Failed Immunotherapy	Unknown	Melanoma Stage Iv Unresectable Stage III Melanoma	Procedure: Fecal microbiome Transplant (FMT)	Phase 1

Probiotics are healthy, living bacteria that can be used as adjuvant therapy in tumor treatment to enhance immunity. Probiotics have been shown to regulate intestinal microflora and have demonstrated antitumor activity in several studies.^153^, ^260^ Animal study results showed that *Lactobacillus acidophilus* and *Bifidobacterium* upregulated miR‐26b and miR18a and downregulated miR135b, miR155, and KRAS expression, inhibiting colon cancer progression.^155^ Clinical results show that probiotics can maintain intestinal flora balance after CRC surgery and effectively protect the intestinal mucosal barrier, prevent postoperative inflammation, reduce postoperative complications, and reduce the incidence of diarrhea caused by 5‐fluorouracil and postoperative radiotherapy.^156^ Probiotics derived from *Akkermansia muciniphila* (*A. muciniphila*) are considered to be promising candidates.^261^ Four metastatic melanoma patients whose immunotherapy for anti‐PD‐1 led to clinical response were found to have abundant *A. muciniphila* .^262^ Also, Routy et al. investigated the relationship between immunocheckpoint inhibitors and gut microbiota in cancer patients. According to their findings, patients receiving PD‐1 antibodies reported a significantly higher intestinal level of *A. muciniphila*.^263^ *A. muciniphila* multiplied in response to the applied density.^264^ Based on these findings, it can be concluded that cancer immunotherapy in combination with *A. muciniphila* as one of the important probiotics in selective microbiota transplantation will produce better outcomes for patients in the near future.^265^, ^266^ Accordingly, it is reported one patient with high‐grade metastatic urothelial carcinoma experienced ICIs‐associated colitis following combined CTLA‐4 and CTLA‐3 therapies. The patient's intestinal tract showed evidence of good colonization of donor‐derived bacteria after treatment with FMT for ICI‐associated colitis, as evidenced by the presence of a higher level of *A. muciniphila*.^267^

Prebiotics support selective fermentation including oligomers of fructose (FOS), xylan, galactose (GOS), inulin, and fructan. Prebiotic compounds can increase the proportion of beneficial intestinal bacteria, stimulate SCFA synthesis, regulate immune responses, modify microbial gene expression, increase cecum and colon absorption of micronutrients, and regulate metabolic enzymes.^25^ It was found that FOS and inulin‐rich prebiotics combined with *Lactobacillus rhamnosus* and *Bifidobacterium lactis* inhibited azoxymethanase‐induced proliferation in rat colon cancer cells.^25^ GOS produced by galactosidase or glucosidase conversion can increase the concentration of intestinal lactate and SCFAs, reduce the concentration of secondary bile acid and lactic acid in the feces, inhibit the activity of intestinal nitroreductase and glucuronidase, and may prevent the occurrence of CRC. Probiotic microorganisms consume prebiotic fibers for energy metabolism and growth. Moreover, prebiotic supplementation resulted in significant increases in IgG and IgM levels in perioperative CRC patients.^268^

**FIGURE 4:**
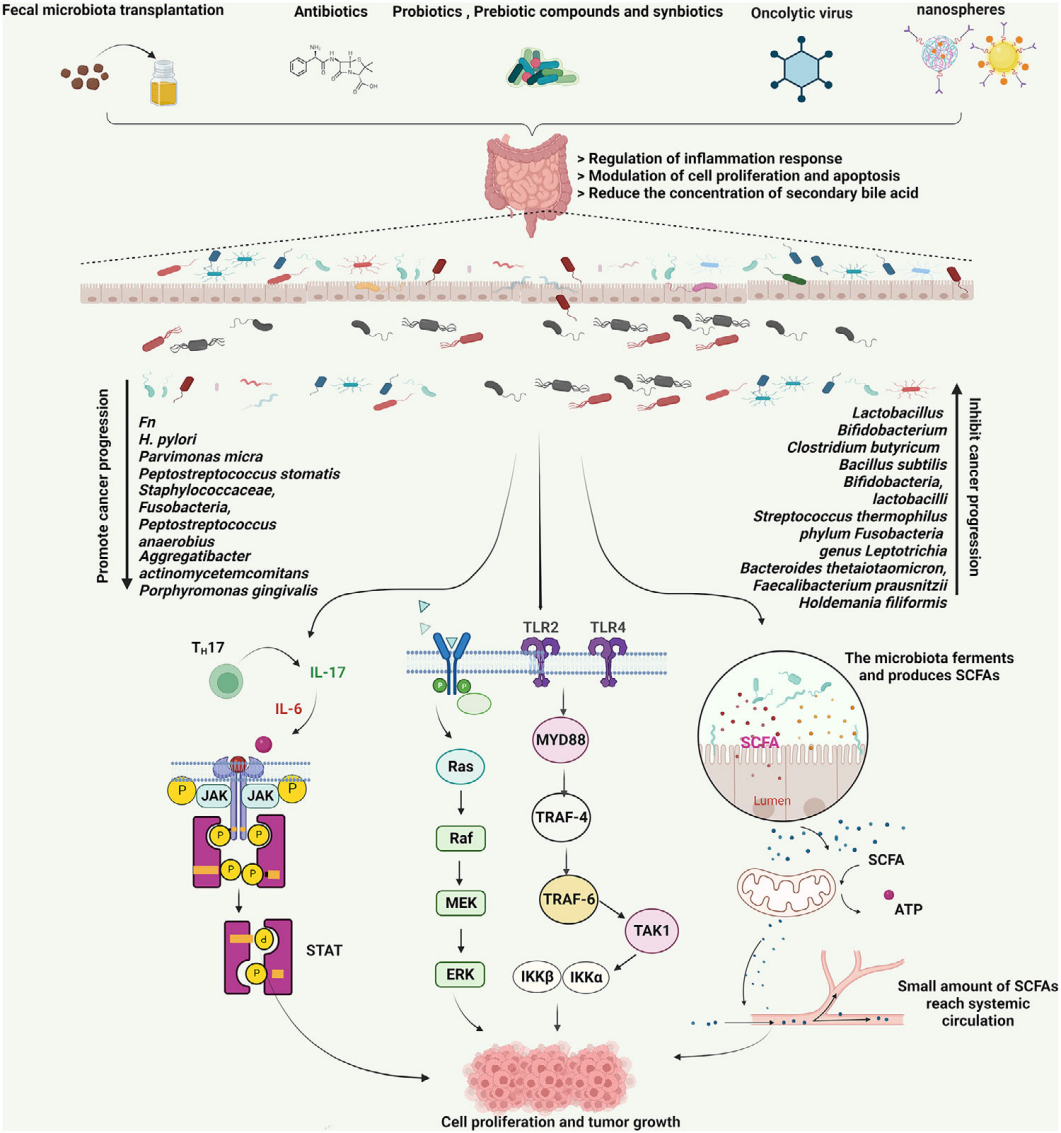
Microbiome treatment for cancer. Possible therapeutic approaches for cancer include fecal microbiome transplantation (FMT), probiotics, prebiotic compounds, synbiotics, antibiotics, oncolyitc virus and nanospheres, which are processes through which the intestinal microbiome can regulate inflammation, modulate cell proliferation and apoptosis, and reduce the concentration of secondary bile acids. By activating the JAK/STAT, PI3K/AKT, Ras/MEK, and TLR2/4‐MYD88 pathways, which are involved in tumorigenesis, microbial dysbiosis and special bacteria can influence cancer development and progression. The microbes that ferment and produce SCFAs also affect cell proliferation and tumor growth. Several microbes, such as *Fn*, *Helicobacter pylori, Fusobacterium, Staphylococcus aureus*, *Peptostreptococcus anaerobius*, and *Porphyromonas gingivalis*, promote cancer progression. Other microbes, such as *Lactobacillus*, *Bifidobacterium*, *Clostridium butyricum*, *Bacillus subtilis*, *Bifidobacteria*, *Lactobacoilli*, *Streptococcus thermophilus*, *Leptotrichia*, *Bacteroides thetaiotaomicron*, and *Faecalibacterium prausnitzii*, can inhibit cancer progression.

Combining probiotics and prebiotics, synbiotics have been used to treat a variety of tumor types, including CRC. Clinical studies show that synbiotics significantly reduce postoperative infection in CRC patients. In addition, the combined supportive treatment regimen of *L. acidophilus*, *L. rhamnosus*, *Lactobacillus casei*, *Bifidobacterium*, and FOS significantly reduced surgical site infections in CRC patients.^26^, ^269^ Animal experiments have confirmed that a cocktail of acidophiles (64 × 1011 CFU) composed of FOS, maltose dextrin, and lactic acid bacteria inhibited tumor growth, promoted apoptosis, and inhibited inflammation^270^ (Figure 4).

Wide‐spectrum antibiotics have adverse effects on patients receiving cancer immunotherapy. Therefore, the use of specific antibiotics may be beneficial to patients. Antibiotics targeting specific microbial flora can regulate immune capacity. For example, depletion of bacteria sensitive to vancomycin enhanced tumor sensitivity to radiation therapy and significantly inhibited tumor growth.^271^, ^272^ Bacteriophages are viruses that can infect and kill bacteria, and they naturally exist in the microbiome where they play a key role in maintaining community balance. Phages can reduce environmental pathogenic bacteria; however, no phage is currently available for the treatment of clinical disease.^273^

In recent years, oncolytic viruses have become an increasingly popular method of treating cancer. *Adenovirus* is the most potent and first virus used in oncolytic virotherapy. Several studies have recently indicated that other viruses can also be candidates for cancer therapies, such as *herpes simplex virus* (*HSV*) and *measles virus*. There has been a successful Phase III clinical trial for *T‐VEC*, an *HSV‐based oncolytic virus* approved by the United States Food and Drug Administration for use in biological cancer therapies. Preclinical and clinical trials have demonstrated impressive results for the vaccine strain of the *measles virus*. With their therapeutic efficacy, safety, and reduced side effects, such engineered viruses may represent a major breakthrough in cancer treatment.^274^ Radiation‐induced antitumor immune responses are regulated by intestinal fungi in breast cancer and melanoma mouse models.^275^

Microbiota intervention techniques, such as antibiotics, probiotics, and microbiota transplantation, have been used traditionally to boost cancer treatments’ efficacy. Moreover, new microbial treatments, such as nanotechnology, have been developed for antitumor therapy. A new approach for tumor therapy is provided by nanotechnology, which alters the microbiome's size and intervenes in microbiological molecules from a microscale perspective.

It is widely known that nanotechnology can be used to treat cancer. The first generation of nanotechnology has numerous advantages for clinical applications. These benefits include to deliver the drug targeted to the correct tissues, control its release, improve the permeability and solubility, improve the effectiveness, and decrease the toxicity of the drug. Second nanotechnology is in the process of being clinically tested. As a result of its current advantages, other functions of nanoscale particles have been enhanced, including targeting tissues, improved drug delivery, and stimulation of the immune system. There are more advantages to nanotechnology in the third generation, such as the regulation of the immune system and the ability to penetrate biological barriers. An anticancer particle based on nanotechnology could enhance the tumor microenvironment, promote local immunity cell proliferation, increase the ability of cancer microorganisms to penetrate tissues, and inhibit their growth. In addition to intervene microbiomes‐tumor microenvironment, Nanotechnology‐based anticancer particle can target cancer‐promoting metabolites secreting by microbiomes and improve locally hypoxic in the tumor microenvironment.

## 5 CONCLUSIONS AND PERSPECTIVE

The human microbiome is a complex network that interacts with the host in multiple body parts. In recent years, the role of the microbiome in disease development has become increasingly important, affecting the development, progression, and metastasis of many types of cancers (Tables 2 and 3).

**TABLE 3:** Human bacteria with putative anticancer properties.

Bacteria	cancer	Molecular mechanism
*Lactobacillus*	Colon cancer	Upregulated caspase‐3, caspase‐9, and Bax and down‐regulated Bcl2, cyclin D1, cyclin E, and ERBB2 genes.^26^, ^269^
*Bifidobacterium*	Colon cancer	Induced the tumor suppressor miRNAs (miR‐145 and miR‐15a) expression.^26^, ^269^
*Clostridium butyricum*	Colon cancer	Inhibited NF‐κB pathway and promoted apoptosis.^115^
*Bacillus subtilis*	Oral cancer	Inhibited PI3K/Akt/ NF‐κB and AP‐1/IL‐6 signaling pathways.^276^
*Bifidobacteria*	Colon cancer	Down‐regulated antiapoptotic genes and upregulated proapoptotic genes.^277^
*Lactobacoilli*	Cervical cancer	Upregulated E‐cadherin.^278^
*Streptococcus thermophilus*	Colorectal cancer	Secreted β‐galactosidase.^279^
*Faecalibacterium prausnitzii*	Colon cancer	Suppressed lipid peroxidation levels.^280^

Recombinant *Listeria monocytogenes* vaccine showed a good inhibitory effect against tumor cell proliferation in mouse. As a kinds of phytotoxins vector, bacteria can harbor ricin, saporin, or pseudomonal exotoxins to suppress protein synthesis, induce cancer cells apopotosis. Investigators struggled with regulating microbiome to enhance cancer treatment response and eliminate toxic side effects of cancer therapy. Numerous studies indicated that interaction between external (diet, antigen exposure, drug, and mental stress) and host inherent factors can regulate cancer‐promoting microbioma and exert an antitumor effect. Fecal microbiota transplantation, probiotics, next‐generation biotherapeutics, designer microbial consortia, and targeting the tumor microbiota are commonly performed therapeutic for the management of cancer. However, the complex of the microbiomes promoting cancer mechanisms is still far from being completely understood. For example, there is a widely bidirectional feedback between microbiomes and neuroendocrine system. The microbiomes anticancer exact mechanism is unclear. Moreover, commercially available probiotic formulas generally is not clear. There are also differences in reactivity to tumor suppression. Commercially available probiotic can also have adverse effects such as diarrhea.

However, this is still a novel and young field. Many questions remain to open especially the exact anticancer mechanism of a certain microbioma. It is important to clarify the complex ecosystem in cancer development and is involved in many aspects of tumorigenesis from basic, epidemiological and clinical research. Consequently, it is imperative to understand how microorganisms and their secretions contribute to cancer occurrence and progression. Their advantages and limitations in the treatment of tumors could help to elucidate the mechanisms underlying tumor occurrence and progression. It will provide new strategies for the development of microbial‐related personalized drugs to improve treatment efficacy.

## AUTHOR CONTRIBUTION

C. L. X., J. Y. S., C. L., Z. K. M., S. H. Y., and C. S. Y. collected the related reports. C. L. X. drafted the manuscript. J. Y. S., C. L., L. W. F., and C. L. X. revised the manuscript. L. W. F. and C. L. X. participated in designing the review. All authors read and approved the final manuscript.

## CONFLICT OF INTEREST STATEMENT

The authors declare no conflicts of interest.

## ETHICS STATEMENT

Not applicable.

## Data Availability

Not applicable.
